# A comparison of comorbidities and their risk factors prevalence across rheumatoid arthritis, psoriatic arthritis and axial spondyloarthritis with focus on cardiovascular diseases: data from a single center real-world cohort

**DOI:** 10.1007/s00296-024-05740-z

**Published:** 2024-11-11

**Authors:** Zofia Guła, Katarzyna Łosińska, Piotr Kuszmiersz, Magdalena Strach, Jarosław Nowakowski, Grzegorz Biedroń, Olena Zimba, Łukasz Dyczek, Glenn Haugeberg, Mariusz Korkosz

**Affiliations:** 1 Department of Rheumatology, Immunology and Internal Medicine, University Hospital in Krakow, Krakow, Poland; 2 Department of Rheumatology and Immunology, Jagiellonian University Medical College, Kraków, Poland; 3 National Institute of Geriatrics, Rheumatology and Rehabilitation, Warsaw, Poland; 4 Department of Internal Medicine N2, Danylo Halytsky Lviv National Medical University, Lviv, Ukraine; 5 Division of Rheumatology, Department of Internal Medicine, Sørlandet Hospital, Kristiansand, Norway; 6 Department of Neuromedicine and Movement Science, Faculty of Medicine and Health Sciences, NTNU, Norwegian University of Science and Technology, Trondheim, Norway

**Keywords:** Rheumatoid arthritis, Psoriatic arthritis, Axial spondyloarthritis, Comorbidities, Cardiovascular diseases, Risk factors, Hypertension, Dyslipidemia, Obesity, Osteoporosis, Depression, Inflammatory arthritis, Real-world data.comorbidities, Inflammatory arthritis, Real-world evidence, Cardiovascular risk

## Abstract

Management of comorbidities is essential to a patient-centered approach to the treatment of chronic inflammatory arthritis. The aim of this study was to compare the prevalence of comorbidities and their risk factors in rheumatoid arthritis (RA), psoriatic arthritis (PsA), and axial spondyloarthritis (axSpA) in a single center outpatient cohort. This cross-sectional study included adult patients diagnosed with RA, PsA, and axSpA from a single rheumatology outpatient center. Comorbidities were documented by physicians, and patients were categorized into two age groups, younger (< 45 years) and older (≥ 45 years), with age- and gender-based comparisons. Disease activity, comorbidities, and cardiovascular (CV) risk factors were analyzed using chi-squared tests for categorical variables and independent samples t-tests for continuous variables, with *p* values < 0.05 considered statistically significant. Comorbidities were registered by physicians using GoTreatIt® Rheuma software. Among 508 RA, 267 PsA, and 285 axSpA patients, the four most common comorbidities were hypertension (36.4%, 25.1%, and 19.7%, respectively), dyslipidemia (19.5%, 15.4%, 14.7% respectively), obesity (16.9%, 22.5%, 14% respectively) and thyroid disease (21.5%, 13.9%, 11.2% respectively). Other comorbidities differed among the diseases and included osteoporosis, osteoarthritis, diabetes mellitus, arrhythmia, and asthma in RA, diabetes mellitus, depression and asthma in PsA, osteoporosis and serious infection in axSpA. RA patients, compared to axSpA had a higher prevalence of coronary artery disease (4.1% vs. 0.7%, *p* = 0.006), arrhythmia (6.9% vs. 2.5%, *p* = 0.008) and major adverse cardiac events (2.6% vs. 0.4%, *p* = 0.024) compared to axSpA. Osteoporosis was more frequent in RA (19.1%) and axSpA (8.4%) than in PsA (2.3%; *p* < 0.001) and was frequently diagnosed in patients aged < 45. Depression prevalence was surprisingly low (1.6%, 5.2%, and 1.8%, respectively). RA patients had the highest multimorbidity rate, with 26.6% reporting three or more comorbidities, compared to 16.8% in PsA and 10.6% in axSpA (*p* < 0.001). Health status was poorest in RA and worse in women compared to men for all diseases. RA, PsA, and axSpA share the same four most common comorbidities: hypertension, dyslipidemia, obesity, and thyroid disease but have different prevalence of other disorders and CV risk factors, indicating the need for an individual screening and prevention approach. The possible unrecognition of depression should be evaluated.

**Supplementary Information:**

The online version contains supplementary material available at 10.1007/s00296-024-05740-z.

## Introduction

Patient-centred care in rheumatology requires detecting and optimising the management of concomitant disorders. Comorbidities influence both the activity and variety of outcomes in rheumatic disease, have an impact on the effectiveness and adverse events of different pharmacological agents and increase socioeconomic cost on an individual and systemic level [1–5]. European League Against Rheumatism (EULAR) recommends screening for six comorbidities in daily rheumatological practice: cardiovascular ischemic diseases, infections, cancers, osteoporosis, gastrointestinal diseases, and depression [6]. However, implementation of such a strategy is often limited due to the lack of practical tools for detecting and reporting non-rheumatic disorders and the lack of integration within medical records. This results in divergent data on epidemiology, structure, and the impact of comorbidities in inflammatory rheumatic diseases. Moreover, some important comorbidities, such as fibromyalgia, are not included in EULAR recommendations [6].

In eastern Europe, the profile of comorbidities associated with inflammatory arthritis presents unique challenges due to specific demographic trends and healthcare practices. The regional healthcare system, while comprehensive, faces barriers to the integration of multidisciplinary care, which is essential for managing complex cases of inflammatory arthritis. The Polish population exhibits certain lifestyle and genetic predispositions that may influence the prevalence and impact of comorbidities. Understanding these variances is crucial to tailoring interventions that may improve patient outcomes and reduce the burden on the healthcare system [7].

The aim of this study is to comprehensively compare the prevalence of comorbidities and associated risk factors among patients with the three most common types of inflammatory arthritis—rheumatoid arthritis (RA), psoriatic arthritis (PsA), and axial spondyloarthritis (axSpA)—in a real-world outpatient setting. This study not only focuses on the overall disease population but also considers differences based on age and sex, thereby offering comorbidity patterns in younger (< 45 years) and older (≥ 45 years) populations. Special attention is given to cardiovascular diseases (CVD) and other clinically significant comorbidities, such as osteoporosis, thyroid diseases, and depression, as these conditions heavily influence patient management and long-term outcomes.

## Materials and methods

### Study design

This study was designed as a cross-sectional, observational study, conducted at the rheumatology outpatient clinic of The University Hospital in Krakow, Poland, involving adult patients involving adult patients, conducted at the rheumatology outpatient clinic of the University Hospital in Krakow, Poland.

### Inclusion and exclusion criteria

To be included in the study, participants were required to be over 18 years old and have a confirmed diagnosis based on established criteria: Rheumatoid Arthritis (RA) diagnosed according to the 2010 American College of Rheumatology/European League Against Rheumatism (ACR/EULAR) classification criteria [8], Psoriatic Arthritis (PsA) diagnosed either in its axial or peripheral form as per the Classification Criteria for Psoriatic Arthritis (CASPAR) [9], and Axial Spondyloarthritis (axSpA) identified using the Assessment of SpondyloArthritis international Society (ASAS) criteria [10]. Eligible patients had to attend a rheumatology outpatient clinic and have their data recorded in the GoTreatIt® Rheuma software [11] during routine clinical care. It was necessary for the clinical records to contain sufficient data, including demographic information, disease activity scores, comorbidity profiles, and treatment regimens. Patients were required to provide written informed consents for the use of their clinical data in the study.

The study excluded patients who did not have a confirmed diagnosis of RA, PsA, or axSpA according to the specified classification criteria. Additionally, patients whose medical records lacked essential information on comorbidities, disease activity, or cardiovascular risk factors, which made a comprehensive analysis impossible, were not included. Lastly, patients who declined to provide written informed consents for the use of their clinical data or withdrew their consents during the study were not considered.

### Data collection and data variables

Data collection was recorded at clinical visits as a part of routine practice with a frequency specified by the treating doctor. Recommended outcome measures were collected and followed using the GoTreatIt^®^ Rheuma software as part of standard clinical care [11]. Data extraction was performed on November 6, 2023. A predefined query was used to retrieve data from the hospital database and displayed data structured on Excel datasheets.

Demographic variables included age, sex, body mass index (BMI, kg/m2), smoking status, physical activity level and disease duration. C-reactive protein (CRP), Health Assessment Questionnaire (HAQ),36-Item 36-item Short Form Survey (SF-36) and the following composite scores were assessed: Disease Activity Score in 28 joint counts with CRP (DAS28-CRP), Disease Activity Index for Psoriatic Arthritis (DAPSA), Bath Ankylosing Spondylitis Disease Activity Index (BASDAI), Ankylosing Spondylitis Disease Activity Score (ASDAS). Current treatment, including non-steroidal anti-inflammatory drugs (NSAIDs), conventional synthetic DMARDs (csDMARDs), biological/targeted synthetic DMARDs (b/tsDMARDs) and glucocorticoids (GCs) was recorded. Demographic data HAQ and SF-36 were self-registered by the patients. Standardized joint counts were performed by doctors and collected along with laboratory and treatment data. Comorbidity data were collected methodically through a dual approach: initially, patients provided information about their comorbid conditions through a structured questionnaire during their visit, which was followed by a thorough review of their electronic health records (EHR) to confirm these conditions. Subsequently, the treating physicians conducted detailed evaluations of the patients' treatment histories to further validate the presence and management of these comorbidities. Apart from all group comparisoncomparisons, we separately analyzed data for younger (< 45 years old) and older (≥ 45 years old) patients. Major adverse cardiac events (MACE) included cardiac death, non-fatal myocardial infarction (MI) and stroke. Because cardiovascular (CV) risk increases with age [12], we separately analyzed younger and older populationthe younger and older populations of patients. We arbitrarily chose 45 years as a cut-off point, which is in accordance with the entrance criterion of lower back pain in the ASAS classification criteria of axSpA [10]. Notably, we intended to find whether a younger population is at risk of CVD, without already diagnosed coronary artery disease or MACE.

### Statistical analyzes

Absolute and/or relative frequencies were used to describe the population means and standard deviations (SD). Chi-squared for categorical variables and independent samples t-test for continuous variables were used to compare patient characteristics.

All statistical analyzes were performed on available data with no imputation of missing data in SAS Studio (SAS Institute, Cary, North Carolina, USA). *P*-values < 0.05 were considered significant.

### Ethics approval and patient involvement

This research is a part of the Polish-Norwegian “POLNOR-RHEUMA” project dedicated to enhancing the healthcare quality and improving health outcomes of patients with rheumatic diseases in Poland [13]. The study was approved by the Bioethics Committee of the Jagiellonian University Medical College (protocol N 1072.6120.247.2020) and adhered to the principles of the Declaration of Helsinki. Written informed consents were obtained from all patients. Patients were not involved in this study's design, conduct, reporting, or dissemination.

## Results

### Demographics, disease activity and treatment in RA, PsA, and axSpA

The characteristics of the patients and the overall prevalence of comorbidities across all diagnoses are shown in Table 1. Patients with RA were the oldest and had the longest duration of the disease compared to patients with PsA and axSpA (mean age in RA versus PsA versus axSpA: 56.9 vs. 48.8 vs. 42.9 years; mean duration of the disease 11.4 vs. 8 vs. 8.1 years, respectively). Women constituted 79.1% of RA patients, compared to 54.7% of PsA and 44.9% of axSpA. Treatment modalities were different in RA vs. PsA vs. axSpA.: Current use of csDMARD was 67.3%, 52.1%, and 6%, respectively; bDMARD 33.1%, 39% and 64.6%, respectively; JAK inhibitors: 11.2% vs. 7.5% and 3.5%, respectively; GCs: 29.1% vs. 8.6% and 1.1%, respectively; NSAIDs: 28.9%, 35.2% vs. 55.8% respectively.

**Table 1:** Patients characteristic, disease related parameters and comorbidities across RA, PsA and axSpA

				*P*-value		
	RA (n = 508)	PsA (n = 267)	axSpA (n = 285)	RA versus PsA	RA versus axSpA	PsA versus axSpA
Age, years, mean (SD) *% missing*	56.9 (14.6) *0.0*	48.8 (13.0) *0.0*	42.9 (12.3) *0.0*	< 0.001	< 0.001	< 0.001
BMI kg/m^2^ mean (SD)	26.0 (5.1) *15.6*	27.7 (5.4) *12.0*	25.7 (4.6) *5.6*	< 0.001	0.49	< 0.001
Disease duration, years, mean (SD)	11.4 (8.7) *9.3*	8.0 (8.0) *11.2*	8.1 (7.7) *6.7*	< 0.001	< 0.001	0.80
DAS28-CRP mean (SD)	3.5 (1.6) *6.7*	3.1 (1.6) *8.6*	NA	0.006	NA	NA
DAPSA mean (SD)	NA	16.4 (15.0) *18.4*	NA	NA	NA	NA
BASDAI mean SDI)	NA	3.5 (2.4) *62.9*	2.7 (2.2) *0.4*	NA	NA	0.002
ASDAS mean (SD)	NA	1.8 (0.9) *82.8*	1.6 (1.0) *20.7*	NA	NA	0.27
CRP mg/l, mean (SD)	6.0 (12.5) *5.1*	5.7 (9.6) *4.5*	5.0 (11.8) *6.7*	0.80	0.31	0.44
HAQ mean (SD)	1.0 (0.7) *9.5*	0.8 (0.7) *4.9*	0.5 (0.6) *1.4*	0.007	< 0.001	< 0.001
SF36_MH	59.3 (17.5) *12.8*	61.2 (18.0) *13.1*	59.0 (16.4) *14.0*	0.19	0.82	0.17
SF36_VT	47.2 (18.9) *12.8*	49.3 (18.6) *13.9*	46.6 (17.5) *13.7*	0.18	0.67	0.10
SF36_BP	41.2 (24.6) *12.8*	41.7 (26.5) *12.4*	42.6 (25.4) *14.0*	0.80	0.45	0.68
SF36_GH	36.6 (15.8) *13.4*	39.3 (17.9) *12.7*	38.8 (15.0) *14.0*	0.06	0.07	0.78
SF36_SF	61.1 (24.6) *12.6*	63.0 (24.0) *12.4*	62.6 (23.9) *13.7*	0.34	0.44	0.86
SF36_PF	57.1 (26.3) *12.6*	60.2 (25.7) *12.7*	58.8 (25.8) *14.4*	0.14	0.42	0.54
SF36_RP	37.2 (41.1) *13.0*	41.2 (42.7) *13.5*	38.0 (40.8) *13.7*	0.24	0.81	0.4
SF36_RE	54.9 (44.3) *12.8*	58.3 (45.1) *13.1*	57.4 (43.4) *14.4*	0.34	0.47	0.81
SF36_HT	45.3 (30.0) *12.0*	45.3 (30.9) *12.4*	44.6 (29.4) *13.7*	0.98	0.79	0.80
Sex (M)	106 (20.9) *0.0*	121 (45.3) *0.0*	157 (55.1) *0.0*	< 0.001	< 0.001	0.022
Smoking (ever)	222 (51.4) *15.0*	105 (45.1) *12.7*	117 (43.7) *6.0*	0.12	0.047	0.75
Physical activity (none)	328 (67.9) *4.9*	176 (67.7) *2.6*	120 (42.6) *1.1*	0.07	< 0.001	< 0.001
csDMARDs (now)	342 (67.3) *0.0*	139 (52.1) *0.0*	17 (6.0) *0.0*	< 0.001	< 0.001	< 0.001
bDMARDs (now)	168 (33.1) *0.0*	104 (39.0) *0.0*	184 (64.6) *0.0*	0.10	< 0.001	< 0.001
bDMARDs (ever)	209 (41.1) *0.0*	125 (46.8) *0.0*	203 (71.2) *0.0*	0.13	< 0.001	< 0.001
NSAIDs (now)	147 (28.9) *0.0*	94 (35.2) *0.0*	159 (55.8) *0.0*	0.07	< 0.001	< 0.001
GCs (now)	148 (29.1) *0.0*	23 (8.6) *0.0*	3 (1.1) *0.0*	< 0.001	< 0.001	< 0.001
GCs (ever)	229 (45.1) *0.0*	45 (16.9) *0.0*	14 (4.9) *0.0*	< 0.001	< 0.001	< 0.001
JAKi (now)	57 (11.2) *0.0*	20 (7.5) *0.0*	10 (3.5) *0.0*	0.10	< 0.001	0.039
JAKi (ever)	84 (16.5) *0.0*	26 (9.7) *0.0*	13 (4.6) *0.0*	0.01	< 0.001	0.018
Hypertension	185 (36.4) *0.0*	67 (25.1) *0.0*	56 (19.7) *0.0*	0.001	< 0.001	0.13
Heart failure	11 (2.2) *0.0*	2 (0.8) *0.0*	3 (1.1) *0.0*	0.15	0.25	0.71
Arrhythmia	35 (6.9) *0.0*	9 (3.4) *0.0*	7 (2.5) *0.0*	0.04	0.008	0.52
Coronary artery disease	21 (4.1) *0.0*	5 (1.9) *0.0*	2 (0.7) *0.0*	0.10	0.006	0.22
Myocardial infarction	9 (1.8) *0.0*	3 (1.1) *0.0*	1 (0.4) *0.0*	0.49	0.09	0.29
Stroke	4 (0.8) *0.0*	2 (0.8) *0.0*	0 (0.0) *0.0*	0.95	0.13	0.14
MACE	13 (2.6) *0.0*	5 (1.9) *0.0*	1 (0.4) *0.0*	0.55	0.024	0.09
Pulmonary embolism	5 (1.0) *0.0*	1 (0.4) *0.0*	1 (0.4) *0.0*	0.36	0.32	0.96
Deep vein thrombosis	2 (0.4) *0.0*	1 (0.4) *0.0*	2 (0.7) *0.0*	0.10	0.56	0.6
Obesity	86 (16.9) *15.6*	60 (22.5) *12.0*	40 (14.0) *5.6*	0.06	0.29	0.010
Dyslipidemia	99 (19.5) *0.0*	41 (15.4) *0.0*	42 (14.7) *0.0*	0.16	0.09	0.84
Diabetes mellitus	42 (8.3) *0.0*	21 (7.9) *0.0*	7 (2.5) *0.0*	0.85	0.001	0.004
Thyroid disease	109 (21.5) *0.0*	37 (13.9) *0.0*	32 (11.2) *0.0*	0.01	< 0.001	0.35
Gastric ulcer	8 (1.6) *0.0*	4 (1.5) *0.0*	8 (2.8) *0.0*	0.94	0.24	0.29
Gastroesophageal reflux disease	6 (1.2) *0.0*	5 (1.9) *0.0*	8 (2.8) *0.0*	0.44	0.10	0.47
Liver disease	11 (2.2) *0.0*	10 (3.8) *0.0*	5 (1.8) *0.0*	0.2	0.69	0.15
Cholecystic disease	13 (2.6) *0.0*	1 (0.4) *0.0*	4 (1.4) *0.0*	0.03	0.28	0.20
Asthma	32 (6.3) *0.0*	14 (5.2) *0.0*	13 (4.6) *0.0*	0.56	0.31	0.71
Chronic obstructive pulmonary disease	9 (1.8) *0.0*	1 (0.4) *0.0*	2 (0.7) *0.0*	0.10	0.22	0.60
Interstitial lung disease	10 (2.0) *0.0*	0 (0.0) *0.0*	1 (0.4) *0.0*	0.021	0.06	0.33
Serious infection	22 (4.3) *0.0*	9 (3.4) *0.0*	16 (5.6) *0.0*	0.52	0.42	0.21
Herpes zoster infection	2 (0.4) *0.0*	0 (0.0) *0.0*	0 (0.0) *0.0*	0.31	0.29	NA
Hepatitis type B and/or C	7 (1.4) *0.0*	1 (0.4) *0.0*	3 (1.1) *0.0*	0.19	0.69	0.35
Tuberculosis	1 (0.2) *0.0*	1 (0.4) *0.0*	1 (0.4) *0.0*	0.64	0.68	0.96
Osteoporosis	97 (19.1) *0.0*	6 (2.3) *0.0*	24 (8.4) *0.0*	< 0.001	< 0.001	0.001
Solid cancer	20 (3.9) *0.0*	9 (3.4) *0.0*	8 (2.8) *0.0*	0.69	0.41	0.70
Leukaemia/lymphoma	5 (1.0) *0.0*	0 (0.0) *0.0*	1 (0.4) *0.0*	0.1	0.32	0.33
Psychiatric disorder	21 (4.1) *0.0*	28 (10.5) *0.0*	17 (6.0) *0.0*	0.001	0.25	0.05
Depression	8 (1.6) *0.0*	14 (5.2) *0.0*	5 (1.8) *0.0*	0.004	0.85	0.025
Fibromyalgia	11 (2.2) *0.0*	12 (4.5) *0.0*	10 (3.5) *0.0*	0.07	0.26	0.55
Anaemia	20 (3.9) *0.0*	4 (1.5) *0.0*	3 (1.1) *0.0*	0.06	0.02	0.64
Neutropenia	1 (0.2) *0.0*	0 (0.0) *0.0*	0 (0.0) *0.0*	0.47	0.45	NA
Allergy	9 (1.8) *0.0*	4 (1.5) *0.0*	5 (1.8) *0.0*	0.78	0.99	0.81
Chronic kidney disease	15 (3.0) *0.0*	9 (3.4) *0.0*	7 (2.5) *0.0*	0.75	0.68	0.52
Osteoarthritis	47 (9.3) *0.0*	13 (4.9) *0.0*	12 (4.2) *0.0*	0.03	0.009	0.71
ORL disease	16 (3.2) *0.0*	7 (2.6) *0.0*	4 (1.4) *0.0*	0.68	0.13	0.31
RDCI	0.86 (1.12) *0.0*	0.64 (1.03) *0.0*	0.51 (0.86) *0.0*	0.008	< 0.001	0.102

*ASDAS* Ankylosing Spondylitis Disease Activity Score, *BASDAI* Bath Ankylosing Spondylitis Disease Activity Index, *BMI* body mass index, *CRP* C-reactive protein, *csDMARDs* conventional synthetic DMARDs, *DAPSA* Disease Activity Index for Psoriatic Arthritis, *DAS28* Disease Activity Score in 28 joint counts, *DMARDs* disease modifying antirheumatic drugs, *GCs* glucocorticosteroids, *HAQ* Health Assessment Questionnaire, *JAKi* Janus kinase inhibitors, *MACE* major adverse cardiovascular events, *NSAIDs* non-steroidal anti-inflammatory drugs, *ORL* othorhinophalangeal, *SD* standard deviation, *SF-36* 36-Item Short Form Survey

### Most common comorbidities across RA, PsA, and axSpA

The four most common comorbidities in RA, PsA, and axSpA were the following (Table 1): hypertension (36.4%, 25.1%, and 19.7%, respectively), dyslipidemia (19.5%, 15.4%, and 14.7%, respectively), obesity (16.9%, 22.5%, and 14%, respectively), and thyroid disease (21.5%, 13.9%, and 11.2%, respectively).

In RA, other most prevalent concomitant disorders were osteoporosis (19.1%), osteoarthritis (9.2%), diabetes mellitus (8.3%), arrhythmia (6.9%), asthma (6.3%), serious infection (4.3%), and coronary artery disease (4.1%). In PsA, respectively: diabetes mellitus (7.9%), depression (5.2%), asthma (5.2%), osteoarthritis (4.9%), fibromyalgia (4.5%), liver disease (3.8%) and chronic kidney disease (3.4%). In axSpA, the most common subsequent comorbidities were osteoporosis (8.4%), serious infection (5.6%), asthma (4.6%), osteoarthritis (4.2%), fibromyalgia (3.5%), gastric ulcer (2.9%), and gastroesophageal reflux disease (2.9%).

Significant differences in the prevalence of these comorbidities across diagnoses (Table 1) include osteoporosis (RA > axSpA > PsA), osteoarthritis (RA > PsA = axSpA), thyroid disease (RA > PsA > axSpA), diabetes mellitus (RA = PsA > axSpA), depression (PsA > RA = axSpA).

**Fig. 1:**
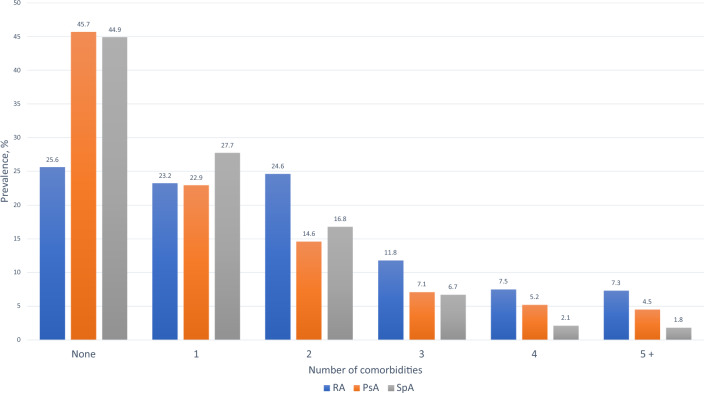
Number of concomitant disorders in RA, PsA and axSpA

**Fig. 2:**
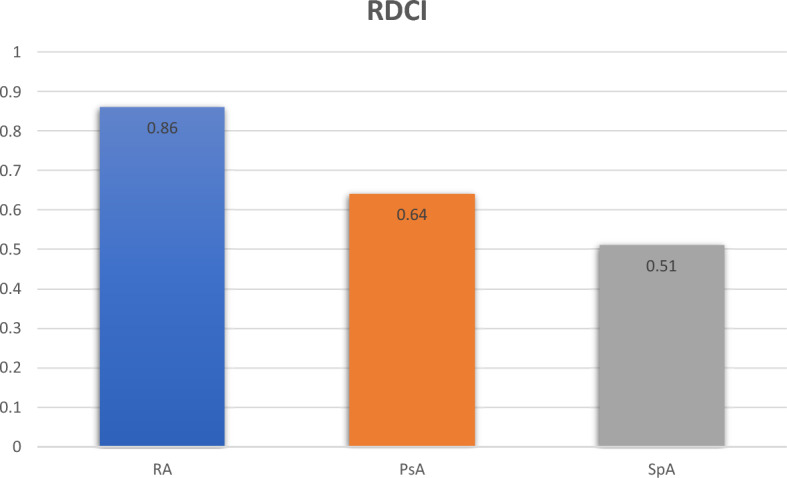
Rheumatic disease comorbidity index (RDCI) across RA, PsA and axSpA

We also found differences in the number of all concomitant disorders (Fig. 1) and in comorbidity burden measured by Rheumatic Disease Comorbidity Index (RDCI) [14] (Fig. 2), comparable in PsA and axSpA and higher in RA. Patients free of any comorbidities were more frequent among PsA (45.7%) and axSpA (44.9%) than among RA (25.6%). Three or more comorbidities were reported in 26.6% of patients with RA, 16.8% of PsA, and only 10.6% of patients with axSpA.

### Prevalence of cardiovascular diseases and profiles of CVD risk factors

RA patients, compared to axSpA, had a significantly higher prevalence of coronary artery disease (4.1% vs. 0.7%, *P* = 0.006), arrhythmia (6.9% vs. 2.5%, *P* = 0.008), and MACE (2.6% vs. 0.4%, *P* = 0.024). The prevalence of these conditions in PsA was 1.9%, 3.4%, and 1.9%, respectively. We also observed significant differences in several CVD risk factors (Table 1). Importantly, RA patients were the oldest, and had the longest disease duration and highest exposure to GCs.

#### Prevalence of cardiovascular disease in the group of patients < 45 years

The prevalence of CVD disease was low and similar in the three groups of patients (Table S1). No coronary artery disease and MACE cases were reported in the younger population. There was one case of pulmonary embolism in PsA and 2 cases of deep vein thrombosis in axSpA.

The most frequent CVD risk factors in RA versus PsA versus axSpA were: hypertension (9%, 12%, 8%, respectively), dyslipidemia (5%, 12%, 11%, respectively), obesity (10.9%, 20.4%, 12.9%, respectively), diabetes mellitus (3%, 1%, 2%, respectively), history of smoking (40%, 36%, 44%, respectively), no physical activity (59%, 66%, 35%, respectively), current use of NSAIDs (26%, 35%, 54%, respectively) and exposure to GCs (40%, 16%, 5%, respectively).

We found significant differences between the three groups in terms of traditional CV risk factors as well as disease and treatment-related CV risk factors. PsA patients had a higher BMI and, more often, no physical activity than patients with RA and axSpA. RA patients had a longer duration of the disease and a higher exposure to GCs, and SpA patients had higher exposure to NSAIDs.

#### Prevalence of cardiovascular disease in the group of patients ≥ 45 years old

Compared to younger patients, those ≥ 45 years of age had a higher prevalence of cardiovascular diseases and MACE (Table S2), without significant differences between the three groups (despite arrhythmia, more frequent in RA than in axSpA). The reported prevalence of CVD in RA, PsA and axSpA: heart failure (2.3%, 0.7% and 1.8%, respectively), arrhythmia (7.8%, 3.9%, and 1.8%, respectively), coronary artery disease (5.3%, 3.3%, and 1.8%, respectively), myocardial infarction (2.3%, 2%, and 0.9%, respectively), stroke (1%, 1.3%, and 0%, respectively). No death from CVD was observed in our cohort. Pulmonary embolism was reported in 5 patients with RA and 1 patient with axSpA, while DVT was reported in 2 patients with RA and 1 patient with PsA.

We also observed higher prevalence of CV risk factors: hypertension (44%, 34.4%, 37.7% respectively), dyslipidemia (23.6%, 18.2%, 20.2% respectively), obesity (18.6%, 24%, 15.8% respectively), diabetes mellitus (9.8%, 12.3%, 2.6% respectively), history of smoking (54.8%, 51.9%, 43.2% respectively), no physical activity (70.4%, 69.3%, 53.5% respectively), current use of NSAIDs (29.9%, 35.7%, 57.9% respectively) and exposure to GCs (46.5%, 17.5%, 5.3% respectively).

Some of these risk factors significantly differed across the diagnoses: RA patients were the oldest, had a longer duration of the disease than PsA patients, a higher rate of smoking than axSpA, and a higher prevalence of hypertension than PsA patients. Patients with PsA had the highest BMI. Both RA and PsA patients, compared to axSpA, were diagnosed with diabetes mellitus more frequently and had no physical activity. In terms of treatment, axSpA were significantly more frequently treated with NSAIDs, while exposure to GCs was higher in RA, than in PsA, and least frequent in axSpA.

### Sex comparison of disease activity and comorbidities in RA, PsA, and axSpA

The characteristic and comparison of comorbidities in men and women is presented in Table S3. There were no significant differences in the prevalence of CVD and MACE between men and women in all diseases. However, in RA, men were older than women (60 vs 56.1 years old), more frequently smoking (75% vs 45.4%), but had a shorter duration of the disease (9.4 vs 11.9 years). RA disease activity (DAS28-CRP and CRP) was similar in both groups. RA women, compared with men, were more often diagnosed with thyroid disease (23.9 vs 12.3%), asthma (7.5 vs 1.9%), osteoporosis (21.1 vs 11.3%) and any psychiatric disorder (5.2 vs 0%), but lower rate of leukaemia/lymphoma (0.5% vs 2.8%).

PsA women compared with men were older (50.9 vs. 46.2 years), with a comparable duration of the disease but higher disease activity measured by DAS28-CRP (3.6 vs. 2.5), DAPSA (20.4 vs. 11.7) and BASDAI (2.5 vs 2.1), but not ASDAS nor CRP. Women with PsA compared to men had more thyroid disease (31% vs 6%), asthma (12% vs 2%), any psychiatric disorder (16.4% vs. 3.3%) and fibromyalgia (8.2 vs 0%).

Women with axSpA were comparable in age to men but had shorter disease duration (5.3 vs 8.9 years) and higher disease activity: BASDAI (3.2 vs 2.3), ASDAS (1.8 vs 1.5) but similar CRP. They also more frequently had thyroid disease (21.1% vs. 3.2%), gastrointestinal disease (15.6% vs. 6.4%), cholecystic disease (3.1% vs. 0%) and fibromyalgia (6.3 vs 1.3%).

### Health status and quality of life

Interestingly, we found significant differences in health status (Table 1) measured by the Health Assessment Questionnaire (HAQ) in RA, PsA and axSpA (accordingly 1.0 vs. 0.8 vs. 0.5), indicating that RA had the greatest impact on the physical function of patients. Health status was poorer in women than in men (Table S4) in all diseases (HAQ in RA: 1.0 vs. 0.8, in PsA 1.0 vs. 0.6, in axSpA 0.7 vs. 0.4). However, no significant differences were found in all domains of quality of life measured by the SF-36 questionnaire in the three diagnoses and in the comparison of men and women.

## Discussion

In all examined diseases, the leading four comorbidities—hypertension, dyslipidemia, obesity and thyroid disease—were the same, but we found different patterns of other concomitant disorders. The comorbidities with the prevalence of > 5% reported in RA were osteoporosis, osteoarthritis, diabetes mellitus, arrhythmia, and asthma; in PsA: diabetes mellitus, depression, and asthma; and in axSpA: osteoporosis and serious infections.

RA patients, compared to axSpA, had a higher prevalence of coronary artery disease, arrhythmia, and MACE (non-fatal myocardial infarction and stroke combined, with no case of CVD death), which may be explained by a higher rate of traditional as well as treatment and disease-related risk factors, notably older age, more frequent hypertension, smoking and diabetes mellitus, longer duration of the disease, and greater exposure to GCs. Interestingly, the prevalence of cardiovascular diseases was comparable in patients with RA and PsA, possibly due to a similar metabolic burden in these conditions, especially the substantial prevalence of diabetes mellitus.

However, the CVD risk factors profiles in respective IA were slightly different. As mentioned above, RA patients were overall older and had a longer disease duration, had hypertension more frequently, and had the highest exposure to GCs. PsA patients had a higher rate of metabolic disorders – higher BMI and more frequent diabetes. Compared to RA and PsA, patients with axSpA were more exposed to NSAIDs. Interestingly, axSpA patients seem to have more physical activity than patients with RA and PsA.

Only a few studies directly compare concomitant disorders in RA, PsA, and axSpA, mostly focused on cardiovascular diseases [15, 16]. It is however unclear whether the screening strategies should be the same or disease-specific, considering differences in inflammatory pathways, proportions of traditional risk factors, and treatment modalities [17]. EULAR recommends a multiplication factor of 1.5 to calculate CV risk scores only in patients with RA [18], and no multiplication factor is approved for PsA and axSpA. The 2021 update of the European Society of Cardiology (ESC) recommendations for CV prevention suggests that patients with a broad spectrum of chronic inflammatory diseases should be considered a general high-risk population [19]. Some country-specific recommendations, i.e., the Joint American Academy of Dermatology (AAD)-National Psoriasis Foundation (NPF) advise the use of a 1.5 multiplier in skin psoriatic patients (PsO) with more severe skin involvement [20], so perhaps the same consideration should be applied in PsA. However, in expert opinion, such a strategy could lead to overtreatment of some patients with PsA, and possibly different multipliers may be required in different clinical settings [17]. Similarly, in axSpA, there is an urgent need for more data on disease specific CV risk factors, thus warranting  the development of respective recommendations [21].

A recent study comparing IA patients with healthy controls found an increased risk of cardiovascular disease in patients with RA (OR 1.61; 95% CI: 1.04 to 2.48) and PsA (2.12; 95% CI: 1.23 to 3.66), and a trend towards increased odds in patients with SpA (OR 1.43; 95% CI: 0.79 to 2.59), attributable to the higher prevalence of traditional risk factors in rheumatic patients [15]. No significant differences in the incidence and prevalence of MACE between RA, PsA, and axSpA were reported in another large cohort of patients, suggesting that inflammation, rather than a particular type of arthritis, drives the increased risk of cardiovascular disease [16]. The direct comparison of RA, PsA, and axSpA in our study seems to be consistent with the position that the risk of CVD and MACE is comparable in RA and PsA and possibly smaller in axSpA. However, given the low prevalence and relatively small group of patients, we are unable to draw a certain conclusion. On the other hand, a different profile of CV risk factors—BMI, physical activity, exposure to NSAIDs and GCs—observed already in the younger population, may be helpful in a further approach to the prevention of CVD development in daily practice on the country-specific level. Rheumatologists should pay even more attention to encouraging patients with RA and PsA to do physical activity and be more vigilant about the risk of diabetes and obesity in these patients, compared to axSpA. The frequency of other risk factors in the younger population, smoking, dyslipidemia, and hypertension, was generally the same, although its quite high prevalence indicates a need for country-specific systemic strategies for screening and controlling these modifiable CV risk factors in the real world.

We also found different profiles other than CVD comorbidities. PsA patients had a significantly lower prevalence of osteoporosis both in the younger and older population. In patients ≥ 45 years, osteoporosis was more common in RA than in axSpA. Although systemic inflammation leading to osteoporosis is present in all three diseases, some specific bone turnover processes are driven by different pathways in RA, PsA and axSpA [22]. The excess of osteoporosis in RA may also be explained by more frequent GCs therapy, older age, longer duration of the disease, and a higher ratio of women to men. Interestingly, there was no difference in the prevalence of osteoporosis between RA and axSpA patients < 45 years old, supporting the hypothesis that osteoporosis occurs at an early stage of axSpA [23, 24]. Therefore, it seems reasonable to screen for osteoporosis even in young patients with axSpA and RA. The lack of healthy control prevented us from concluding whether in PsA screening for osteoporosis should differ from general recommendations. However, the prevalence of this condition was very low in our cohort.

In line with some previous findings [25], PsA patients suffered most frequently from psychiatric disorders and depression. A recent study on > 12,000 patients with IA showed that the prevalence of depression was 18.61% in RA, 27.2% in PsA and 26.4% in SpA [26]. Although these data differ from the pooled frequencies reported in the meta-analysis (RA: 32.1% [1], PsA: 11.9% [2], axSpA: 10.9% [3]), depression is one of the most common comorbidities in IA. The surprisingly low prevalence of depression in our cohort suggests that it is underrecognized in Polish patients, possibly for several reasons, both patient-related (fear of being stigmatised by a psychiatric disorder, downplaying low mood) and systemic (difficulties in accessing psychiatrists, lack of time and skills to suspect depression during regular rheumatologic appointments). It strengthens the need for practical tools for the real-life evaluation of mood disorders in Polish patients with IA, which is essential to the effective and comprehensive treatment of these diseases.

In our cohort, patients without any comorbidity were found in only 25.6% of RA, compared to 45.7% of PsA and 44.9% of axSpA, which is most likely related to older age, longer duration of the disease, and greater exposure to GCs in RA patients. In line with these data, RDCI, encompassing lung disease, CVD (heart attack, other cardiovascular disease, or stroke), hypertension, fracture, depression, diabetes, cancer, ulcer or stomach symptoms, was higher in RA than in PsA and axSpA, indicating higher burden of comorbidities in RA. We also found the worst health status measured by HAQ in patients with RA, which is consistent with previous data [27] and supports the evidence of a substantial impact of multimorbidity on health status in rheumatic inflammatory diseases [28, 29]. Additionally, in line with previous data, we found that physical function was worse in women than in men [30]. Remarkably, these differences were not reflected in the quality of life of our patients, since all SF-36 domains, including physical function in SF-36, were comparable in men and women across all studied diseases. It underlines that the comprehensive assessment of a wide variety of patient-reported outcome measures (PROMs) in chronic arthritis provides a more accurate picture of the disease and may indicate that coping and adaptation mechanisms during chronic illness are at play.

The strength of our study is the direct comparison of the three most frequent IA, using the same structured electronic database and recording every comorbidity in  a real-world setting by health professionals. Also, the results became the basis for drawing practical conclusions in everyday work, e.g. the need for early detection of osteoporosis in younger patients and screening for depression. The limitations of this study include the absence of healthy controls and cross-sectional design, which precludes a search for a causal relationship between the presence of risk factors and the occurrence of particular concomitant diseases. However, this is the first Polish study systematically assessing comorbidities in rheumatic patients. We provided real-world data on the prevalence of comorbidities, with a focus on CVD in the three most common inflammatory arthritis, also taking into account age and sex differences. To our knowledge, this is the first such analysis in Poland, which is based on the quality registry of rheumatic diseases managed at the Jagiellonian University in Krakow. In the future, such a structural approach to collecting data on rheumatic disease, comorbidities, and PROMs could be supportive in developing country-specific recommendations for risk stratification and prevention of comorbidities in daily practice, ultimately contributing to more comprehensive and patient-centred healthcare.

In conclusion, apart from the four most common concomitant disorders (hypertension, dyslipidemia, obesity and thyroid disease), we found different patterns of other comorbidities in RA, PsA, and axSpA, indicating a need for a more individual approach to screening procedures, i.e. detection of osteoporosis already in younger patients with RA and axSpA. The high prevalence of traditional as well as treatment and disease-related modifiable CVD risk factors requires a well-thought-out prevention strategy. The possible underdiagnosis of depression in Polish patients needs further evaluation and improved detection.

## Supplementary Information

Below is the link to the electronic supplementary material.

Supplementary file1 (DOCX 53 kb)

## Data Availability

Open data sharing All data are available from the corresponding author upon a reasonable request.
