# Supplementary material for: A comparison of comorbidities and their risk factors prevalence across rheumatoid arthritis, psoriatic arthritis and axial spondyloarthritis with focus on cardiovascular diseases: data from a single center real-world cohort
Source: Rheumatol Int. 2024 Nov 11;44(12):2817–28. doi: 10.1007/s00296-024-05740-z (PMC11618134; doi:10.1007/s00296-024-05740-z)
Supplement: Supplementary file 1 — Supplementary file1 (DOCX 53 kb) [file 296_2024_5740_MOESM1_ESM.docx]

Table S1. Patients characteristic, disease related parameters and comorbidities across RA, PsA and axSpA in the group <45 years old

|  | | | | | ***P*-value** | | | |
| --- | --- | --- | --- | --- | --- | --- | --- | --- |
|  |  |  |  |  |  | |  |  |
|  | **RA (n=110)** | **PsA (n=113)** | **axSpA (n=171)** | **RA vs PsA** | | **RA vs axSpA** | | **PsA vs axSpA** |
| Age, years, mean (SD) | 35.4 (6.9) | 37.0 (6.1) | 34.6 (6.3) | 0.07 | | 0.34 | | 0.002 |
| BMI kg/m^2,^ mean (SD) | 24.1 (4.9) | 27.4 (6.0) | 25.5 (4.8) | <0.001 | | 0.023 | | 0.008 |
| Disease duration mean, years (SD) | 9.0 (6.8) | 6.2 (6.3) | 6.0 (5.1) | 0.003 | | <0.001 | | 0.83 |
| DAS28-CRP mean (SD) | 3.0 (1.6) | 2.7 (1.6) | NA | 0.13 | | NA | | NA |
| DAPSA mean (SD) | NA | 13.1 (13.6) | NA | NA | | NA | | NA |
| BASDAI mean SDI) | NA | 3.3 (2.4) | 2.6 (2.2) | NA | | NA | | 0.040 |
| ASDAS mean (SD) | NA | 1.7 (0.9) | 1.5 (1.0) | NA | | NA | | 0.48 |
| CRP mean (SD) mg/l | 5.2 (11.7) | 5.8 (9.8) | 5.6 (14.1) | 0.67 | | 0.83 | | 0.84 |
| HAQ mean (SD) | 0.8 (0.7) | 0.6 (0.7) | 0.4 (0.5) | 0.17 | | <0.001 | | 0.009 |
|  |  |  |  |  | |  | |  |
| SF36_MH | 59.6 (19.1) | 59.5 (18.3) | 58.5 (16.5) | 0.99 | | 0.64 | | 0.65 |
| SF36_VT | 46.3 (18.8) | 48.5 (20.2) | 46.1 (16.9) | 0.43 | | 0.93 | | 0.32 |
| SF36_BP | 42.5 (26.6) | 41.7 (27.6) | 43.1 (24.8) | 0.84 | | 0.86 | | 0.69 |
| SF36_GH | 37.9 (17.1) | 37.5 (19.6) | 40.3 (15.4) | 0.89 | | 0.26 | | 0.25 |
| SF36_SF | 58.4 (23.9) | 62.1 (24.8) | 62.9 (23.2) | 0.29 | | 0.14 | | 0.79 |
| SF36_PF | 63.9 (24.0) | 62.5 (25.2) | 59.9 (25.2) | 0.68 | | 0.21 | | 0.44 |
| SF36_RP | 38.5 (41.1) | 43.2 (42.1) | 36.9 (41.2) | 0.44 | | 0.76 | | 0.26 |
| SF36_RE | 58.3 (43.3) | 57.2 (46.3) | 59.7 (42.4) | 0.86 | | 0.81 | | 0.67 |
| SF36_HT | 44.4 (28.4) | 46.1 (32.2) | 45.4 (28.9) | 0.70 | | 0.80 | | 0.85 |
|  | | | | | | | | |
| Sex (M) | 16 (14.6) | 61 (54.0) | 99 (57.9) | <0.001 | | <0.001 | | 0.52 |
| Smoking (ever) | 40 (40.0) | 36 (36.0) | 69 (44.0) | 0.56 | | 0.53 | | 0.21 |
| Physical activity (none) | 64 (59.3) | 72 (65.5) | 59 (35.1) | 0.036 | | <0.001 | | <0.001 |
| csDMARDs (now) | 70 (63.6) | 52 (46.0) | 8 (4.7) | 0.008 | | <0.001 | | <0.001 |
| bDMARDs (now) | 52 (47.3) | 51 (45.1) | 107 (62.6) | 0.75 | | 0.012 | | 0.004 |
| bDMARDs (ever) | 56 (50.9) | 60 (53.1) | 119 (69.6) | 0.74 | | 0.002 | | 0.005 |
| NSAIDs (now) | 28 (25.5) | 39 (34.5) | 93 (54.4) | 0.14 | | <0.001 | | 0.001 |
| Steroids (now) | 24 (21.8) | 5 (4.4) | 2 (1.2) | <0.001 | | <0.001 | | 0.08 |
| Steroids (ever) | 44 (40.0) | 18 (15.9) | 8 (4.7) | <0.001 | | <0.001 | | 0.001 |
| JAKi (now) | 11 (10.0) | 11 (9.7) | 6 (3.5) | 0.95 | | 0.026 | | 0.030 |
| JAKi (ever) | 19 (17.3) | 14 (12.4) | 6 (3.5) | 0.31 | | <0.001 | | 0.004 |
|  |  |  |  |  | |  | |  |
| Hypertension | 10 (9.1) | 14 (12.4) | 13 (7.6) | 0.43 | | 0.66 | | 0.18 |
| Heart failure | 2 (1.8) | 1 (0.9) | 1 (0.6) | 0.55 | | 0.33 | | 0.77 |
| Arrhythmia | 4 (3.6) | 3 (2.7) | 5 (2.9) | 0.67 | | 0.74 | | 0.89 |
| Coronary artery disease | 0 (0.0) | 0 (0.0) | 0 (0.0) | NA | | NA | | NA |
| Myocardial infarction | 0 (0.0) | 0 (0.0) | 0 (0.0) | NA | | NA | | NA |
| Stroke | 0 (0.0) | 0 (0.0) | 0 (0.0) | NA | | NA | | NA |
| MACE | 0 (0.0) | 0 (0.0) | 0 (0.0) | NA | | NA | | NA |
| Pulmonary embolism | 0 (0.0) | 1 (0.9) | 0 (0.0) | 0.32 | | NA | | 0.22 |
| Deep vein thrombosis | 0 (0.0) | 0 (0.0) | 2 (1.2) | NA | | 0.26 | | 0.25 |
| Obesity | 12 (10.9) | 23 (20.4) | 22 (12.9) | 0.05 | | 0.62 | | 0.09 |
| Dyslipidemia | 5 (4.6) | 13 (11.5) | 19 (11.1) | 0.06 | | 0.06 | | 0.92 |
| Diabetes mellitus | 3 (2.7) | 2 (1.8) | 4 (2.3) | 0.63 | | 0.84 | | 0.74 |
| Thyroid disease | 21 (19.1) | 11 (9.7) | 19 (11.1) | 0.046 | | 0.06 | | 0. 71 |
| Gastric ulcer | 0 (0.0) | 1 (0.9) | 5 (2.9) | 0.32 | | 0.07 | | 0.24 |
| GERD | 2 (1.8) | 1 (0.9) | 3 (1.8) | 0.55 | | 0.97 | | 0.54 |
| Liver disease | 0 (0.0) | 2 (1.8) | 2 (1.2) | 0.16 | | 0.26 | | 0.67 |
| Cholecystic disease | 1 (0.9) | 0 (0.0) | 2 (1.2) | 0.31 | | 0.84 | | 0.25 |
| Asthma | 5 (4.6) | 5 (4.4) | 4 (2.3) | 0.97 | | 0.31 | | 0.33 |
| Chronic obstructive pulmonary disease | 1 (0.9) | 0 (0.0) | 0 (0.0) | 0.31 | | 0.21 | | NA |
| Interstitial lung disease | 1 (0.9) | 0 (0.0) | 0 (0.0) | 0.31 | | 0.21 | | NA |
| Serious infection | 4 (3.6) | 4 (3.5) | 7 (4.1) | 0.97 | | 0.85 | | 0.81 |
| Herpes zoster infection | 0 (0.0) | 0 (0.0) | 0 (0.0) | NA | | NA | | NA |
| Hepatitis type B and/or C | 1 (0.9) | 1 (0.9) | 1 (0.6) | 0.99 | | 0.75 | | 0.77 |
| Tuberculosis | 0 (0.0) | 0 (0.0) | 0 (0.0) | NA | | NA | | NA |
| Osteoporosis | 6 (5.5) | 1 (0.9) | 11 (6.4) | 0.050 | | 0.74 | | 0.023 |
| Solid cancer | 2 (1.8) | 1 (0.9) | 2 (1.2) | 0.55 | | 0.65 | | 0.82 |
| Leukaemia/lymphoma | 1 (0.9) | 0 (0.0) | 0 (0.0) | 0.31 | | 0.21 | | NA |
| Psychiatric disorder | 4 (3.6) | 9 (8.0) | 9 (5.3) | 0.17 | | 0.53 | | 0.36 |
| Depression | 1 (0.9) | 3 (2.7) | 3 (1.8) | 0.33 | | 0.56 | | 0.61 |
| Fibromyalgia | 2 (1.8) | 6 (5.3) | 3 (1.8) | 0.16 | | 0.97 | | 0.09 |
| Anaemia | 8 (7.3) | 1 (0.9) | 2 (1.2) | 0.015 | | 0.007 | | 0.82 |
| Neutropenia | 1 (0.9) | 0 (0.0) | 0 (0.0) | 0.31 | | 0.21 | | NA |
| Allergy | 2 (1.8) | 2 (1.8) | 3 (1.8) | 0.98 | | 0.97 | | 0.99 |
| Chronic kidney disease | 0 (0.0) | 5 (4.4) | 1 (0.6) | 0.026 | | 0.42 | | 0.028 |
| Osteoarthritis | 4 (3.6) | 1 (0.9) | 3 (1.8) | 0.17 | | 0.32 | | 0.54 |
| ORL disease | 1 (0.9) | 2 (1.8) | 1 (0.6) | 0.55 | | 0.75 | | 0.34 |
| RDCI | 0.29 (0.75) | 0.33 (0.70) | 0.28 (0.61) | 0.707 | | 0.844 | | 0.505 |

ASDAS: Ankylosing Spondylitis Disease Activity Score, BASDAI: Bath Ankylosing Spondylitis Disease Activity Index, BMI: body mass index, CRP: C-reactive protein, csDMARDs: conventional synthetic DMARDs, DAPSA: Disease Activity Index for Psoriatic Arthritis, DAS28: Disease Activity Score in 28 joint counts, DMARDs: disease modifying antirheumatic drugs, GCs: glucocorticosteroids, HAQ: Health Assessment Questionnaire, JAKi: Janus kinase inhibitors, MACE: major adverse cardiovascular events, NSAIDs: non-steroidal anti-inflammatory drugs, ORL: othorhinophalangeal, SD: standard deviation, SF-36: 36-Item Short Form Survey

Table S2. Patients characteristic, disease related parameters and comorbidities across RA, PsA and axSpA in the group ≥ 45 years old

|  | | | | | ***P*-value** | | |
| --- | --- | --- | --- | --- | --- | --- | --- |
|  |  |  |  |  |  |  |  |
|  | **RA (n=398)** | **PsA (n=154)** | **axSpA (n=114)** | **RA vs PsA** | | **RA vs axSpA** | **PsA vs axSpA** |
| Age, years, mean (SD) | 62.9 (9.8) | 57.5 (9.1) | 55.4 (7.7) | <0.001 | | <0.001 | 0.05 |
| BMI kg/m^2^ , mean (SD) | 26.5 (5.0) | 28.0 (4.9) | 26.0 (4.4) | 0.004 | | 0.30 | 0.001 |
| Disease duration, years, mean (SD) | 12.0 (9.0) | 9.3 (8.9) | 11.1 (9.6) | 0.003 | | 0.34 | 0.14 |
| DAS28-CRP mean (SD) | 3.6 (1.5) | 3.4 (1.5) | NA | 0.31 | | NA | NA |
| DAPSA mean (SD) | NA | 18.9 (15.5) | NA | NA | | NA | NA |
| BASDAI mean SDI) | NA | 3.7 (2.4) | 2.9 (2.2) | NA | | NA | 0.043 |
| ASDAS mean (SD) | NA | 1.8 (1.0) | 1.7 (0.9) | NA | | NA | 0.47 |
| CRP mean (SD) mg/l | 6.2 (12.7) | 5.7 (9.5) | 4.2 (7.2) | 0.62 | | 0.040 | 0.17 |
| HAQ mean (SD) | 1.0 (0.7) | 0.9 (0.7) | 0.7 (0.6) | 0.30 | | <0.001 | 0.003 |
|  |  |  |  |  | |  |  |
| SF36_MH | 59.2 (17.0) | 62.3 (17.8) | 59.7 (16.3) | 0.08 | | 0.82 | 0.24 |
| SF36_VT | 47.5 (19.0) | 49.8 (17.5) | 47.2 (18.3) | 0.21 | | 0.92 | 0.27 |
| SF36_BP | 40.8 (24.1) | 41.6 (25.9) | 42.0 (26.4) | 0.73 | | 0.65 | 0.91 |
| SF36_GH | 36.3 (15.5) | 40.5 (16.6) | 36.9 (14.4) | 0.01 | | 0.71 | 0.08 |
| SF36_SF | 61.9 (24.7) | 63.6 (23.6) | 62.1 (25.0) | 0.48 | | 0.92 | 0.65 |
| SF36_PF | 55.1 (26.6) | 58.6 (26.1) | 57.2 (26.6) | 0.19 | | 0.50 | 0.67 |
| SF36_RP | 36.9 (41.1) | 39.9 (43.2) | 39.6 (40.3) | 0.47 | | 0.56 | 0.95 |
| SF36_RE | 53.9 (44.6) | 59.1 (44.5) | 54.1 (44.7) | 0.25 | | 0.96 | 0.39 |
| SF36_HT | 45.5 (30.4) | 44.8 (30.0) | 43.6 (30.1) | 0.81 | | 0.58 | 0.76 |
|  | | | | | | | |
| Sex (M) | 90 (22.6) | 60 (39.0) | 58 (50.9) | <0.001 | | <0.001 | 0.05 |
| Smoking (ever) | 182 (54.8) | 69 (51.9) | 48 (43.2) | 0.57 | | 0.035 | 0.18 |
| Physical activity (none) | 264 (70.4) | 104 (69.3) | 61 (53.5) | 0.16 | | 0.003 | 0.001 |
| csDMARDs (now) | 272 (68.3) | 87 (56.5) | 9 (7.9) | 0.009 | | <0.001 | <0.001 |
| bDMARDs (now) | 116 (29.2) | 53 (34.4) | 77 (67.5) | 0.23 | | <0.001 | <0.001 |
| bDMARDs (ever) | 153 (38.4) | 65 (42.2) | 84 (73.7) | 0.42 | | <0.001 | <0.001 |
| NSAIDs (now) | 119 (29.9) | 55 (35.7) | 66 (57.9) | 0.19 | | <0.001 | <0.001 |
| Steroids (now) | 124 (31.2) | 18 (11.7) | 1 (0.9) | <0.001 | | <0.001 | 0.001 |
| Steroids (ever) | 185 (46.5) | 27 (17.5) | 6 (5.3) | <0.001 | | <0.001 | 0.003 |
| JAKi (now) | 46 (11.6) | 9 (5.8) | 4 (3.5) | 0.044 | | 0.011 | 0.38 |
| JAKi (ever) | 65 (16.3) | 12 (7.8) | 7 (6.1) | 0.009 | | 0.006 | 0.60 |
|  | | | | | | | |
| Hypertension | 175 (44.0) | 53 (34.4) | 43 (37.7) | 0.041 | | 0.23 | 0.58 |
| Heart failure | 9 (2.3) | 1 (0.7) | 2 (1.8) | 0.20 | | 0.74 | 0.40 |
| Arrhythmia | 31 (7.8) | 6 (3.9) | 2 (1.8) | 0.10 | | 0.021 | 0.31 |
| Coronary artery disease | 21 (5.3) | 5 (3.3) | 2 (1.8) | 0.31 | | 0.11 | 0.45 |
| Myocardial infarction | 9 (2.3) | 3 (2.0) | 1 (0.9) | 0.82 | | 0.35 | 0.48 |
| Stroke | 4 (1.0) | 2 (1.3) | 0 (0.0) | 0.77 | | 0.28 | 0.22 |
| MACE | 13 (3.3) | 6 (3.9) | 1 (0.9) | 0.72 | | 0.17 | 0.17 |
| Pulmonary embolism | 5 (1.3) | 0 (0.0) | 1 (0.9) | 0.16 | | 0.74 | 0.24 |
| Deep vein thrombosis | 2 (0.5) | 1 (0.7) | 0 (0.0) | 0.83 | | 0.45 | 0.39 |
| Obesity | 74 (18.6) | 37 (24.0) | 18 (15.8) | 0.15 | | 0.49 | 0.10 |
| Dyslipidemia | 94 (23.6) | 28 (18.2) | 23 (20.2) | 0.17 | | 0.44 | 0.68 |
| Diabetes mellitus | 39 (9.8) | 19 (12.3) | 3 (2.6) | 0.38 | | 0.014 | 0.004 |
| Thyroid disease | 88 (22.1) | 26 (16.9) | 13 (11.4) | 0.17 | | 0.011 | 0.21 |
| Gastric ulcer | 8 (2.0) | 3 (2.0) | 3 (2.6) | 0.96 | | 0.69 | 0.71 |
| Gastroesophageal reflux disease | 4 (1.0) | 4 (2.6) | 5 (4.4) | 0.16 | | 0.015 | 0.42 |
| Liver disease | 11 (2.8) | 8 (5.2) | 3 (2.6) | 0.16 | | 0.94 | 0.30 |
| Cholecystic disease | 12 (3.0) | 1 (0.7) | 2 (1.8) | 0.10 | | 0.47 | 0.40 |
| Asthma | 27 (6.8) | 9 (5.8) | 9 (7.9) | 0.69 | | 0.68 | 0.51 |
| Chronic obstructive pulmonary disease | 8 (2.0) | 1 (0.7) | 2 (1.8) | 0.26 | | 0.86 | 0.40 |
| Interstitial lung disease | 9 (2.3) | 0 (0.0) | 1 (0.9) | 0.06 | | 0.35 | 0.24 |
| Serious infection | 18 (4.5) | 5 (3.3) | 9 (7.9) | 0.50 | | 0.16 | 0.09 |
| Herpes zoster infection | 2 (0.5) | 0 (0.0) | 0 (0.0) | 0.38 | | 0.45 | NA |
| Hepatitis type B and/or C | 6 (1.5) | 0 (0.0) | 2 (1.8) | 0.13 | | 0.85 | 0.10 |
| Tuberculosis | 1 (0.3) | 1 (0.7) | 1 (0.9) | 0.49 | | 0.35 | 0.83 |
| Osteoporosis | 91 (22.9) | 5 (3.3) | 13 (11.4) | <0.001 | | 0.007 | 0.008 |
| Solid cancer | 18 (4.5) | 8 (5.2) | 6 (5.3) | 0.74 | | 0.74 | 0.98 |
| Leukaemia/lymphoma | 4 (1.0) | 0 (0.0) | 1 (0.9) | 0.21 | | 0.90 | 0.24 |
| Psychiatric disorder | 17 (4.3) | 19 (12.3) | 8 (7.0) | 0.001 | | 0.23 | 0.15 |
| Depression | 7 (1.8) | 11 (7.1) | 2 (1.8) | 0.001 | | 0.10 | 0.042 |
| Fibromyalgia | 9 (2.3) | 6 (3.9) | 7 (6.1) | 0.29 | | 0.036 | 0.40 |
| Anaemia | 12 (3.0) | 3 (2.0) | 1 (0.9) | 0.49 | | 0.20 | 0.48 |
| Neutropenia | 0 (0.0) | 0 (0.0) | 0 (0.0) | NA | | NA | NA |
| Allergy | 7 (1.8) | 2 (1.3) | 2 (1.8) | 0.70 | | 0.10 | 0.76 |
| Chronic kidney disease | 15 (3.8) | 4 (2.6) | 6 (5.3) | 0.50 | | 0.48 | 0.26 |
| Osteoarthritis | 43 (10.8) | 12 (7.8) | 9 (7.9) | 0.29 | | 0.37 | 0.98 |
| ORL disease | 15 (3.8) | 5 (3.3) | 3 (2.6) | 0.77 | | 0.56 | 0.77 |
| RDCI | 1.02 (1.15) | 0.88 (1.16) | 0.87 (1.04) | 0.191 | | 0.207 | 0.953 |

ASDAS: Ankylosing Spondylitis Disease Activity Score, BASDAI: Bath Ankylosing Spondylitis Disease Activity Index, BMI: body mass index, CRP: C-reactive protein, csDMARDs: conventional synthetic DMARDs, DAPSA: Disease Activity Index for Psoriatic Arthritis, DAS28: Disease Activity Score in 28 joint counts, DMARDs: disease modifying antirheumatic drugs, GCs: glucocorticosteroids, HAQ: Health Assessment Questionnaire, JAKi: Janus kinase inhibitors, MACE: major adverse cardiovascular events, NSAIDs: non-steroidal anti-inflammatory drugs, ORL: othorhinophalangeal, SD: standard deviation, SF-36: 36-Item Short Form Survey

Table S3. Patients characteristic, disease related parameters and comorbidities across RA, PsA and axSpA – sex differences.

|  | **RA**  **n=508** | | | **PsA**  **n=267** | | | | | **axSpA**  **n=285** | | | |
| --- | --- | --- | --- | --- | --- | --- | --- | --- | --- | --- | --- | --- |
|  | **M**  **n=106** | **F**  **n=402** | ***P*-value** | | **M**  **n=121** | **F**  **n=146** | ***P*-value** | **M**  **n=157** | | **F**  **n=128** | ***P*-value** |  |
| Age, years, mean (SD) | 60.0 (13.0) | 56.1 (14.9) | 0.015 | | 46.2 (11.0) | 51.0 (14.0) | 0.002 | 42.4 (11.7) | | 43.6 (13.1) | 0.43 |  |
| BMI kg/m2 mean (SD) | 26.6 (4.1) | 25.8 (5.3) | 0.17 | | 27.6 (4.1) | 27.9 (6.4) | 0.64 | 25.9 (4.1) | | 25.5 (5.2) | 0.34 |  |
| Disease duration, years mean (SD) | 9.4 (7.0) | 11.9 (9.0) | 0.003 | | 8.8 (8.4) | 7.2 (7.6) | 0.13 | 9.7 (8.9) | | 6.1 (5.3) | <0.001 |  |
| DAS28-CRP mean (SD) | 3.4 (1.5) | 3.5 (1.6) | 0.47 | | 2.5 (1.4) | 3.6 (1.5) | <0.001 | NA | | NA | NA |  |
| DAPSA mean (SD) | NA | NA | NA | | 11.7 (11.1) | 20.4 (16.6) | <0.001 | NA | | NA | NA |  |
| BASDAI mean SDI) | NA | NA | NA | | 2.8 (2.1) | 4.2 (2.5) | 0.003 | 2.3 (1.9) | | 3.2 (2.4) | 0.001 |  |
| ASDAS mean (SD) | NA | NA | NA | | 1.5 (0.9) | 2.1 (0.9) | 0.05 | 1.5 (0.9) | | 1.8 (1.0) | 0.019 |  |
| CRP mean (SD) mg/l | 7.3 (12.5) | 5.6 (12.5) | 0.23 | | 5.8 (11.4) | 5.7 (7.8) | 0.96 | 4.8 (9.5) | | 5.3 (14.2) | 0.75 |  |
| HAQ mean (SD) | 0.8 (0.7) | 1.0 (0.7) | 0.029 | | 0.6 (0.6) | 1.0 (0.7) | <0.001 | 0.4 (0.5) | | 0.7 (0.6) | <0.001 |  |
|  |  |  |  | |  |  |  |  | |  |  |  |
| SF36_MH | 59.5 (18.5) | 59.2 (17.2) | 0.88 | | 60.7 (18.3) | 61.5 (17.9) | 0.74 | 58.5 (15.8) | | 59.5 (17.2) | 0.65 |  |
| SF36_VT | 47.1 (19.9) | 47.2 (18.7) | 0.95 | | 48.1 (19.0) | 50.2 (18.3) | 0.40 | 47.0 (16.3) | | 46.0 (18.8) | 0.64 |  |
| SF36_BP | 39.3 (22.7) | 41.6 (25.1) | 0.42 | | 42.0 (26.3) | 41.4 (26.8) | 0.88 | 42.4 (25.1) | | 42.9 (25.9) | 0.87 |  |
| SF36_GH | 35.7 (14.5) | 36.9 (16.2) | 0.53 | | 38.9 (16.8) | 39.6 (18.8) | 0.78 | 39.3 (15.6) | | 38.2 (14.4) | 0.58 |  |
| SF36_SF | 59.8 (22.8) | 61.4 (25.0) | 0.58 | | 62.0 (24.1) | 63.8 (24.0) | 0.58 | 62.1 (23.8) | | 63.2 (24.2) | 0.73 |  |
| SF36_PF | 57.5 (25.2) | 57.0 (26.6) | 0.85 | | 59.2 (25.7) | 61.0 (25.9) | 0.59 | 59.2 (26.4) | | 58.3 (25.1) | 0.79 |  |
| SF36_RP | 34.5 (40.3) | 38.0 (41.3) | 0.46 | | 37.3 (42.1) | 44.5 (43.1) | 0.20 | 35.9 (39.9) | | 40.5 (41.8) | 0.38 |  |
| SF36_RE | 49.1 (33.3) | 56.4 (44.1) | 0.16 | | 55.1 (46.4) | 60.9 (44.1) | 0.33 | 54.6 (43.6) | | 60.9 (43.0) | 0.26 |  |
| SF36_HT | 44.7 (29.2) | 45.4 (30.2) | 0.85 | | 44.1 (30.9) | 46.3 (31.0) | 0.58 | 44.6 (30.5) | | 44.6 (28.1) | 0.99 |  |
|  |  |  |  | |  |  |  |  | |  |  |  |
| Smoking (ever) | 66 (75.0) | 156 (45.4) | <0.001 | | 52 (47.7) | 53 (42.7) | 0.45 | 72 (48.7) | | 45 (37.5) | 0.07 |  |
| Physical activity (none) | 67 (67.7) | 261 (68.0) | 0.61 | | 77 (65.3) | 99 (69.7) | 0.76 | 59 (37.8) | | 61 (48.4) | 0.18 |  |
| csDMARDs (now) | 85 (80.2) | 257 (63.9) | 0.002 | | 55 (45.5) | 84 (57.5) | 0.049 | 7 (4.5) | | 118 (92.2) | 0.23 |  |
| bDMARDs (now) | 29 (27.4) | 139 (34.6) | 0.16 | | 51 (42.2) | 53 (36.3) | 0.33 | 109 (69.4) | | 75 (58.6) | 0.06 |  |
| bDMARDs (ever) | 39 (36.8) | 170 (42.3) | 0.31 | | 62 (51.2) | 63 (43.2) | 0.19 | 116 (73.9) | | 87 (68.0) | 0.27 |  |
| NSAIDs (now) | 34 (32.1) | 113 (28.1) | 0.42 | | 44 (36.4) | 50 (34.3) | 0.72 | 87 (55.4) | | 72 (56.3) | 0.89 |  |
| Steroids (now) | 39 (36.8) | 109 (27.1) | 0.05 | | 5 (4.1) | 18 (12.3) | 0.018 | 1 (0.6) | | 2 (1.6) | 0.45 |  |
| Steroids (ever) | 52 (49.1) | 177 (44.0) | 0.36 | | 14 (11.6) | 31 (21.2) | 0.036 | 6 (3.8) | | 8 (6.3) | 0.35 |  |
| JAKi (now) | 6 (5.7) | 51 (12.7) | 0.042 | | 13 (10.7) | 7 (4.8) | 0.067 | 3 (1.9) | | 7 (5.5) | 0.10 |  |
| JAKi (ever) | 8 (7.6) | 76 (18.9) | 0.005 | | 13 (10.7) | 13 (8.9) | 0.61 | 4 (2.6) | | 9 (7.0) | 0.07 |  |
|  |  |  |  | |  |  |  |  | |  |  |  |
| Hypertension | 43 (40.6) | 142 (35.3) | 0.32 | | 29 (24.0) | 38 (26.0) | 0.70 | 30 (19.1) | | 26 (20.3) | 0.80 |  |
| Heart failure | 5 (4.7) | 6 (1.5) | 0.043 | | 0 (0.0) | 2 (1.4) | 0.20 | 0 (0.0) | | 3 (2.3) | 0.05 |  |
| Arrhythmia | 10 (9.4) | 25 (6.2) | 0.25 | | 3 (2.5) | 6 (4.1) | 0.46 | 4 (2.6) | | 3 (2.3) | 0.91 |  |
| Coronary artery disease | 7 (6.6) | 14 (3.5) | 0.15 | | 3 (2.5) | 2 (1.4) | 0.51 | 2 (1.3) | | 0 (0.0) | 0.20 |  |
| Myocardial infarction | 2 (1.9) | 7 (1.7) | 0.92 | | 2 (1.7) | 1 (0.7) | 0.46 | 1 (0.6) | | 0 (0.0) | 0.37 |  |
| Stroke | 0 (0.0) | 4 (1.0) | 0.30 | | 0 (0.0) | 2 (1.4) | 0.20 | 0 (0.0) | | 0 (0.0) | NA |  |
| MACE | 2 (1.9) | 11 (2.7) | 0.62 | | 2 (1.7) | 4 (2.7) | 0.55 | 1 (0.6) | | 0 (0.0) | 0.37 |  |
| Pulmonary embolism | 1 (0.9) | 4 (1.0) | 0.96 | | 1 (0.8) | 0 (0.0) | 0.27 | 1 (0.6) | | 0 (0.0) | 0.37 |  |
| Deep vein thrombosis | 1 (0.9) | 1 (0.3) | 0.31 | | 0 (0.0) | 1 (0.7) | 0.36 | 1 (0.6) | | 1 (0.8) | 0.89 |  |
| Obesity | 23 (21.7) | 63 (15.7) | 0.14 | | 23 (19.0) | 37 (25.3) | 0.22 | 20 (12.7) | | 20 (15.6) | 0.49 |  |
| Dyslipidemia | 24 (22.6) | 75 (18.7) | 0.36 | | 22 (18.2) | 19 (13.0) | 0.24 | 26 (16.7) | | 16 (12.5) | 0.34 |  |
| Diabetes mellitus | 11 (10.4) | 31 (7.7) | 0.38 | | 11 (9.1) | 10 (6.9) | 0.50 | 2 (1.3) | | 5 (3.9) | 0.15 |  |
| Thyroid disease | 13 (12.3) | 96 (23.9) | 0.010 | | 6 (5.0) | 31 (21.2) | <0.001 | 5 (3.2) | | 27 (21.1) | <0.001 |  |
| Gastric ulcer | 2 (1.9) | 6 (1.5) | 0.77 | | 2 (1.7) | 2 (1.4) | 0.85 | 5 (3.2) | | 3 (2.3) | 0.67 |  |
| Gastroesophageal reflux disease | 1 (0.9) | 5 (1.2) | 0.80 | | 2 (1.7) | 3 (2.1) | 0.81 | 2 (1.3) | | 6 (4.7) | 0.08 |  |
| Liver disease | 2 (1.9) | 9 (2.2) | 0.83 | | 5 (4.1) | 5 (3.4) | 0.76 | 3 (1.9) | | 2 (1.6) | 0.82 |  |
| Cholecystic disease | 1 (0.9) | 12 (3.0) | 0.24 | | 0 (0.0) | 1 (0.7) | 0.36 | 0 (0.0) | | 4 (3.1) | 0.026 |  |
| Asthma | 2 (1.9) | 30 (7.5) | 0.036 | | 2 (1.7) | 12 (8.2) | 0.017 | 6 (3.8) | | 7 (5.5) | 0.51 |  |
| Chronic obstructive pulmonary disease | 3 (2.8) | 6 (1.5) | 0.35 | | 0 (0.0) | 1 (0.7) | 0.36 | 1 (0.6) | | 1 (0.8) | 0.89 |  |
| Interstitial lung disease | 4 (3.8) | 6 (1.5) | 0.13 | | 0 (0.0) | 0 (0.0) | NA | 1 (0.6) | | 0 (0.0) | 0.37 |  |
| Serious Infection | 4 (3.8) | 18 (4.5) | 0.75 | | 5 (4.1) | 4 (2.7) | 0.53 | 6 (3.8) | | 10 (7.8) | 0.15 |  |
| Herpes zoster infection | 0 (0.0) | 2 (0.5) | 0.47 | | 0 (0.0) | 0 (0.0) | NA | 0 (0.0) | | 0 (0.0) | NA |  |
| Hepatitis type B and/or C | 1 (0.9) | 6 (1.5) | 0.67 | | 1 (0.8) | 0 (0.0) | 0.27 | 1 (0.6) | | 2 (1.6) | 0.45 |  |
| Tuberculosis | 0 (0.0) | 1 (0.3) | 0.61 | | 1 (0.8) | 0 (0.0) | 0.27 | 0 (0.0) | | 1 (0.8) | 0.27 |  |
| Osteoporosis | 12 (11.3) | 85 (21.1) | 0.022 | | 2 (1.7) | 4 (2.7) | 0.55 | 12 (7.6) | | 12 (9.4) | 0.60 |  |
| Bone fracture | 0 (0.0) | 10 (2.5) | 0.10 | | 0 (0.0) | 1 (0.7) | 0.36 | 1 (0.6) | | 1 (0.8) | 0.89 |  |
| Solid cancer | 5 (4.7) | 15 (3.8) | 0.64 | | 2 (1.7) | 7 (4.8) | 0.16 | 2 (1.3) | | 6 (4.7) | 0.08 |  |
| Leukaemia/lymphoma | 3 (2.8) | 2 (0.5) | 0.031 | | 0 (0.0) | 0 (0.0) | NA | 1 (0.6) | | 0 (0.0) | 0.37 |  |
| Psychiatric disorder | 0 (0.0) | 21 (5.2) | 0.016 | | 4 (3.3) | 24 (16.4) | 0.001 | 6 (3.8) | | 11 (8.6) | 0.09 |  |
| Depression | 0 (0.0) | 8 (2.0) | 0.14 | | 4 (3.3) | 10 (6.9) | 0.20 | 2 (1.3) | | 3 (2.3) | 0.49 |  |
| Fibromyalgia | 0 (0.0) | 11 (2.7) | 0.09 | | 0 (0.0) | 12 (8.2) | 0.001 | 2 (1.3) | | 8 (6.3) | 0.023 |  |
| Anaemia | 2 (1.9) | 18 (4.5) | 0.22 | | 1 (0.8) | 3 (2.1) | 0.41 | 1 (0.6) | | 2 (1.6) | 0.45 |  |
| Neutropenia | 1 (0.9) | 0 (0.0) | 0.05 | | 0 (0.0) | 0 (0.0) | NA | 0 (0.0) | | 0 (0.0) | NA |  |
| Allergy | 1 (0.9) | 8 (2.0) | 0.47 | | 1 (0.8) | 3 (2.1) | 0.41 | 4 (2.6) | | 1 (0.8) | 0.26 |  |
| Chronic kidney disease | 6 (5.7) | 9 (2.2) | 0.06 | | 5 (4.1) | 4 (2.7) | 0.53 | 3 (1.9) | | 4 (3.1) | 0.51 |  |
| Osteoarthritis | 9 (8.5) | 38 (9.5) | 0.76 | | 3 (2.5) | 10 (6.9) | 0.10 | 5 (3.2) | | 7 (5.5) | 0.34 |  |
| ORL disease | 2 (1.9) | 14 (3.5) | 0.40 | | 5 (4.1) | 2 (1.4) | 0.16 | 3 (1.9) | | 1 (0.8) | 0.42 |  |
| RDCI | 0.88 (1.06) | 0.86 (1.14) | 0.876 | | 0.54 (0.90) | 0.73 (1.12) | 0.122 | 0.41 (0.82) | | 0.63 (0.90) | 0.033 |  |

ASDAS: Ankylosing Spondylitis Disease Activity Score, BASDAI: Bath Ankylosing Spondylitis Disease Activity Index, BMI: body mass index, CRP: C-reactive protein, csDMARDs: conventional synthetic DMARDs, DAPSA: Disease Activity Index for Psoriatic Arthritis, DAS28: Disease Activity Score in 28 joint counts, DMARDs: disease modifying antirheumatic drugs, GCs: glucocorticosteroids, HAQ: Health Assessment Questionnaire, JAKi: Janus kinase inhibitors, MACE: major adverse cardiovascular events, NSAIDs: non-steroidal anti-inflammatory drugs, ORL: othorhinophalangeal, SD: standard deviation, SF-36: 36-Item Short Form Survey
